# The oral microbiome as a regulatory hub for systemic health: a systematic review of mechanistic links and clinical implications

**DOI:** 10.1080/20002297.2026.2635233

**Published:** 2026-03-03

**Authors:** Zhu Ling Guo, Ming Wang Cui, Yu Lei Dong, Shuai Wang

**Affiliations:** a Department of Health Management Center, The First Affiliated Hospital of Hainan Medical University, Haikou, People's Republic of China; b School of Dentistry, Hainan Medical University, Haikou, People's Republic of China; c Department of Stomatology, The Affiliated Hospital of Qingdao University, Qingdao, People's Republic of China

**Keywords:** Oral microbiome, oral dysbiosis, systemic disease, oral-systemic link, inflammation

## Abstract

**Background:**

The human oral microbiome is a highly diverse ecosystem with important roles in oral and systemic health. Beyond dental caries and periodontitis, oral dysbiosis has been increasingly implicated in the development of multiple non-communicable diseases.

**Objective:**

To systematically synthesize evidence on the mechanisms linking oral dysbiosis to systemic diseases and to summarize its diagnostic and therapeutic implications.

**Design:**

A systematic review was performed using major electronic databases. We screened 1,128 records and included 104 studies that met predefined eligibility criteria.

**Results:**

Evidence indicates that oral dysbiosis may influence systemic health through several mechanisms, including hematogenous dissemination of oral pathogens and virulence factors (e.g. lipopolysaccharide), chronic systemic inflammation, molecular mimicry in autoimmune disorders, and microbial metabolic byproducts. The reviewed studies support associations between oral microbiome alterations and atherosclerotic cardiovascular disease, type 2 diabetes, Alzheimer’s disease, rheumatoid arthritis, and gastrointestinal cancers. The literature also highlights the promise of non-invasive oral microbiome-based biomarkers for early detection and disease monitoring. Emerging microbiome-modulating interventions, including probiotics, prebiotics, and bacteriophage therapy, show potential for restoring oral eubiosis and improving systemic outcomes.

**Conclusions:**

Oral dysbiosis is an important regulator of systemic disease processes and a promising target for diagnosis, prevention, and therapy. Integrating oral health and oral microbiome assessment into broader disease management may improve outcomes, although methodological standardization and stronger causal evidence are still needed.

## Graphical abstract

**Figure:**
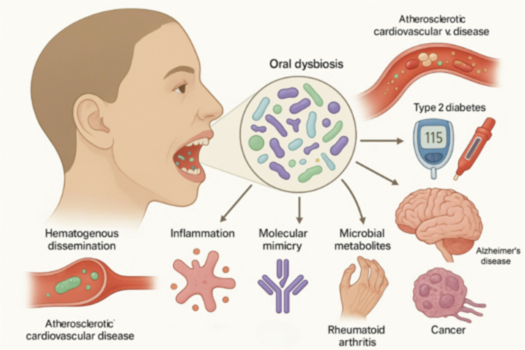

## Introduction

The human body is a superorganism, an intricate symbiosis between host cells and trillions of resident microorganisms. For centuries, the oral cavity was perceived primarily as the portal for nutrition and respiration, with its microbial inhabitants considered relevant only in the context of localised diseases. Pathologies such as dental caries and periodontal disease were, and often still are, treated as isolated conditions, disconnected from the body's overall physiological state. However, this perspective is undergoing a profound transformation, driven by advances in genomic technologies and a deeper understanding of host-microbe interactions. It is now unequivocally clear that the oral cavity is not a sequestered environment but a dynamic and influential gateway to systemic health and disease [1].

The oral microbiome represents the second most diverse and populous microbial community in the human body, surpassed only by the gut. It comprises a highly structured consortium of bacteria, archaea, fungi, and viruses that colonise the various niches of the oral cavity, from the hard surfaces of teeth to the soft mucosal tissues of the tongue and cheeks [2]. This microbial community has co-evolved with its human host over millennia, establishing a delicate equilibrium known as eubiosis, which is fundamental to maintaining oral homoeostasis [3]. In this balanced state, the oral microbiome acts as a protective barrier, preventing the colonisation of exogenous pathogens and actively modulating the local immune system [4].

The disruption of this equilibrium, termed dysbiosis, is the initiating event for the most common oral diseases. Yet, the consequences of oral dysbiosis extend far beyond the confines of the mouth. The ulcerated epithelial surfaces characteristic of periodontal disease provide a chronic portal for oral microbes and their inflammatory byproducts to enter the systemic circulation, thereby influencing distant organ systems [5]. This ‘oral-systemic link’ is no longer a theoretical concept but a field of robust scientific enquiry, supported by a wealth of epidemiological, mechanistic, and clinical evidence. The oral microbiome is now implicated in the initiation or exacerbation of a startling array of systemic conditions, including cardiovascular diseases, metabolic disorders like type 2 diabetes, neurodegenerative diseases, autoimmune conditions, and even certain cancers [6].

This review aims to provide a comprehensive and systematic synthesis of the current evidence establishing the oral microbiome as a central regulatory hub for systemic health. We will begin by defining the composition and ecological principles of the oral microbiome in states of both health (eubiosis) and disease (dysbiosis). Subsequently, we will dissect the core mechanistic pathways through which oral microbes exert their systemic influence. The central portion of this review will critically evaluate the evidence linking the oral microbiome to specific major pathologies, drawing on the most recent research. Finally, we will explore the burgeoning clinical and translational horizons, discussing the potential of the oral microbiome as a source of novel diagnostic biomarkers and as a target for therapeutic interventions designed to improve not only oral health but overall well-being. By integrating these diverse lines of evidence, this review will underscore the clinical imperative to dismantle the artificial barrier between dentistry and medicine and to embrace a more holistic understanding of human health [7].

## Methods

This review was conducted in accordance with the Preferred Reporting Items for Systematic Reviews and Meta-Analyses (PRISMA) guidelines. To ensure the rigour and reproducibility of this systematic review, a comprehensive literature search was conducted across PubMed, Scopus, and Web of Science databases for studies published up to October 2025. The search strategy utilised combinations of MeSH terms and keywords, including ‘oral microbiome,’ ‘periodontitis,’ ‘systemic disease,’ ‘mechanistic pathways,’ and ‘clinical biomarkers.’ Studies were selected based on pre-defined inclusion criteria: (1) peer-reviewed primary research or meta-analyses; (2) studies focusing on the mechanistic links between oral dysbiosis and non-communicable diseases; and (3) clinical trials or longitudinal cohorts involving human oral microbiome data. Exclusion criteria included conference abstracts, case reports, and studies not available in English. Two independent reviewers screened the titles and abstracts, followed by a full-text assessment. Our initial search yielded a total of 1128 records. After removing duplicates and screening titles and abstracts, 186 full-text articles were assessed for eligibility. Ultimately, 104 studies meeting the defined inclusion criteria were selected for this review. The quality and risk of bias of the included studies were assessed to ensure the reliability of the evidence presented. The majority of the included studies demonstrated a moderate to high methodological quality, with a low risk of bias regarding outcome measurement and reporting, thereby supporting the reliability of the synthesised mechanistic evidence (**Supplementary Figure 1**).

## The oral microbiome: a complex ecosystem in health and dysbiosis

### Composition and biogeography of the oral microbiota

The human oral cavity is a remarkably complex and heterogeneous environment, providing a multitude of distinct habitats that support one of the most diverse microbiomes in the body [8]. This intricate ecosystem is composed of a vast array of microorganisms, including bacteria, fungi, archaea, and viruses, which exist in highly structured communities [9]. Over 700 bacterial species have been identified in the oral cavity, though a healthy individual typically harbours around 200 dominant species [10]. The bacterial communities are dominated by six major phyla: Firmicutes, Bacteroidetes, Proteobacteria, Actinobacteria, Spirochaetes, and Fusobacteria, which collectively account for approximately 94% of the oral microbiome [11].

Beyond bacteria, other microbial kingdoms play crucial, albeit less understood, roles. Fungi are consistent members of the healthy oral microbiota, with up to 101 species described, most prominently from the genus *Candida*, but also including *Cladosporium*, *Saccharomyces*, and *Aspergillus* [12]. Archaea, such as *Methanobrevibacter oralis*, represent a minor component but are often found in elevated numbers in subjects with periodontitis [13]. The oral virome, consisting largely of bacteriophages, adds another layer of complexity by modulating bacterial populations and influencing community dynamics.

A defining feature of the oral microbiome is its highly structured biogeography [2]. The oral cavity is not a uniform environment but a collection of distinct microenvironments or niches, each with unique physical and chemical properties. These include hard, non-shedding surfaces like tooth enamel and soft, shedding mucosal surfaces such as the tongue, buccal mucosa, and palate [14]. This topographical diversity drives the formation of distinct microbial communities. For example, streptococci are early colonisers of the tooth surface, creating a foundation for the subsequent attachment of other bacteria, leading to the formation of a complex, multi-species biofilm known as dental plaque [8]. These biofilms are not random aggregations but highly organised structures where metabolic exchange and inter-species communication shape the community's function and pathogenic potential [15]. This intricate organisation underscores the long history of co-evolution between the oral microbiota and its human host, which has resulted in a largely symbiotic relationship under healthy conditions [16].

### The concept of eubiosis and its role in oral homoeostasis

In a state of health, the oral microbiome exists in a state of eubiosis, a dynamic equilibrium characterised by high microbial diversity and a harmonious, symbiotic relationship with the host [10]. This balanced ecosystem is crucial for maintaining oral homoeostasis and performs several vital functions. Much like the commensal microbiota of the skin and gut, the eubiotic oral microbiome forms a protective barrier that resists colonisation by exogenous pathogens through mechanisms such as competitive exclusion for nutrients and attachment sites, and the production of antimicrobial substances like bacteriocins [17].

Furthermore, the commensal microbiota actively engages with and helps to mature the host immune system. It maintains a state of immune tolerance, preventing excessive inflammatory responses to benign stimuli while keeping the local immune system primed to respond to genuine threats [11]. This homoeostatic balance is essential, given that the oral cavity is a primary interface between the external environment and the internal systems of the body, constantly challenged by a barrage of foreign microbes and antigens [8]. The stability of the eubiotic state is therefore a cornerstone of not only oral health but also systemic well-being.

### Drivers of oral dysbiosis: from lifestyle to host genetics

Oral dysbiosis represents a breakdown of this homoeostatic equilibrium. It is not simply an infection by a single pathogen, but rather a community-wide ecological shift that disrupts the balance, often leading to a reduction in beneficial commensal species and an overgrowth of pathobionts—resident microbes with pathogenic potential that are kept in check under eubiotic conditions [18]. This shift creates a pro-inflammatory environment that is conducive to the development of oral diseases like periodontitis and dental caries [1].

The transition from eubiosis to dysbiosis is not a random event but is driven by a complex interplay of environmental, lifestyle, and host-related factors [19]. Key drivers include:

Lifestyle and Diet: A diet high in fermentable carbohydrates, particularly sucrose, provides a selective advantage for acidogenic and acid-tolerant bacteria like *Streptococcus mutans*, driving the dysbiotic process that leads to dental caries [20]. Lifestyle choices such as tobacco smoking and excessive alcohol consumption have also been shown to profoundly alter the composition of the oral microbiome, favoring the growth of anaerobic pathogens and contributing to a pro-inflammatory state [21].

Oral Hygiene: Inadequate oral hygiene allows for the accumulation and maturation of dental plaque. As the biofilm develops, the local environment becomes more anaerobic, favoring the proliferation of Gram-negative anaerobes associated with periodontitis [22].

Host Factors: Systemic conditions can exert a powerful influence on the oral microbiome. For instance, diabetes mellitus creates a hyperglycemic and pro-inflammatory environment in the oral cavity that promotes dysbiosis [23]. The host's immune status and genetic predispositions also play a critical role in shaping the microbial community and the response to it [24].

A crucial concept in understanding the initiation of dysbiosis, particularly in periodontitis, is the ‘keystone pathogen’ hypothesis [25]. This model proposes that certain low-abundance pathogens, most notably *Porphyromonas gingivalis*, can orchestrate a community-wide shift toward disease, even when they constitute a minor component of the total biomass [26].

*P. gingivalis* achieves this by manipulating host immune responses, for example, by subverting complement activation, which in turn alters the local environment to favour the growth of the entire dysbiotic community [27]. This concept represents a significant paradigm shift in microbiology. It moves away from the traditional ‘one pathogen, one disease’ model to an ecological understanding where the functional impact of a single species can reshape the entire community structure and its relationship with the host. This implies that the most effective therapeutic strategies may not be those that aim to reduce the overall bacterial load with broad-spectrum antimicrobials, but rather precision interventions that specifically target the functional activities of these low-abundance keystone orchestrators. Such an approach could involve inhibiting specific virulence factors, like the gingipain proteases of *P. gingivalis*, or using highly selective agents like bacteriophages to remove the keystone species and allow the ecosystem to return to a state of eubiosis.

Furthermore, the multitude of factors influencing the oral microbiome—from diet and smoking to systemic diseases like diabetes and host genetics—underscores that dysbiosis is not a simple infection but a complex ecological collapse [1]. This complexity suggests that a ‘one-size-fits-all’ approach to oral and systemic health is inherently flawed. Clinical management must evolve towards a personalised model that considers an individual's complete lifestyle, genetic background, and systemic health profile, not merely their oral hygiene habits. This aligns with a functional medicine perspective, where oral health is not an isolated discipline but an integral component of a holistic patient assessment, providing a window into their overall physiological state [28].

## Mechanistic pathways linking the oral cavity to systemic circulation

**Figure 1.**
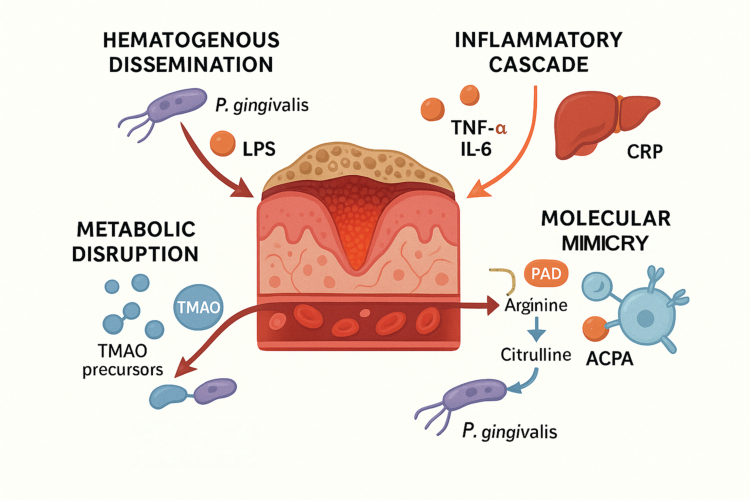
The four primary mechanistic pathways linking oral dysbiosis to systemic disease. From a central site of periodontal inflammation characterised by an ulcerated epithelial barrier, four distinct pathological pathways emanate. (1) Hematogenous dissemination: Oral pathogens such as Porphyromonas gingivalis (*P*. gingivalis) and their virulence factors, like lipopolysaccharide (LPS), enter the systemic circulation. (2) Inflammatory cascade: Locally produced pro-inflammatory cytokines, including Tumour Necrosis Factor-alpha (TNF-*α*) and Interleukin-6 (IL-6), ‘spill over’ into the bloodstream, stimulating the liver to produce C-reactive protein (CRP) and driving a state of chronic, low-grade systemic inflammation. (3) Molecular mimicry: The bacterial peptidylarginine deiminase (PAD) enzyme from *P*. gingivalis converts arginine to citrulline, creating neo-antigens that can lead to the production of anti-citrullinated protein antibodies (ACPAs) and trigger autoimmunity. (4) Metabolic disruption: Microbial metabolites, such as the precursors to the pro-atherogenic molecule trimethylamine *N*-oxide (TMAO), are released into the circulation, contributing to systemic metabolic dysregulation.

The anatomical and physiological connection between the oral cavity and the rest of the body provides multiple pathways for oral microbes and their products to exert systemic effects. The transition from a localised oral inflammatory state to a systemic pathology is not a single event but a complex process involving several interconnected mechanisms. These pathways are not mutually exclusive; rather, they often operate in concert, creating a self-amplifying network that drives chronic disease (Figure 1).

### Hematogenous dissemination: bacteremia and the translocation of microbial products

The oral mucosa, particularly the gingival sulcus, is a highly vascularises tissue. Even routine daily activities such as chewing, tooth brushing, and flossing can cause micro-traumas to this tissue, resulting in transient bacteremia, where viable bacteria from the oral biofilm enter the bloodstream [29]. While these episodes are typically short-lived and cleared by the immune system in healthy individuals, the context changes dramatically in the presence of periodontal disease. Periodontitis is characterised by chronic inflammation and the ulceration of the epithelial lining of the periodontal pockets, creating a persistent and significant portal of entry for a much larger load of bacteria and their virulence factors into the systemic circulation [30].

This hematogenous dissemination is not limited to live bacteria. Crucially, components of the bacterial cell wall, particularly lipopolysaccharide (LPS) from Gram-negative bacteria, are shed into the circulation [31]. LPS, also known as endotoxin, is a potent Pathogen-Associated Molecular Pattern (PAMP) that can trigger strong inflammatory responses throughout the body. The chronic nature of periodontitis means that the body is subjected to a continuous, low-grade endotoxemia originating from the oral cavity [32].

The evidence for this systemic seeding is compelling. Advanced molecular techniques have identified the DNA of oral pathogens in various distant pathological sites. For instance, the DNA of periodontal pathogens such as *Porphyromonas gingivalis*, *Tannerella forsythia*, and *Aggregatibacter actinomycetemcomitans* has been consistently detected within atherosclerotic plaques removed from patients with cardiovascular disease [33]. More acutely, a 2019 study provided the first direct evidence of oral bacterial DNA from *viridans streptococci* in thrombi aspirated from patients experiencing an acute ischaemic stroke, directly linking oral microbes to thromboembolic events [34]. This direct translocation of microbes and their products is a foundational mechanism linking oral disease to systemic inflammation and pathology.

### The inflammatory cascade: local oral inflammation driving systemic responses

Oral dysbiosis, especially in the context of periodontitis, establishes a state of chronic, localised inflammation. The host's immune response to the dysbiotic subgingival biofilm leads to a massive infiltration of immune cells and the local production of a host of pro-inflammatory cytokines, including Tumour Necrosis Factor-alpha (TNF-α), Interleukin-1 beta (IL-1β), and Interleukin-6 (IL-6) [30]. In a contained, acute infection, this response is protective. However, in the chronic setting of periodontitis, this local inflammatory milieu does not remain localised.

The pro-inflammatory mediators produced in the periodontal tissues ‘spill over’ into the systemic circulation, contributing to a state of chronic, low-grade systemic inflammation [35]. This systemic inflammatory burden is a well-established underlying risk factor for a multitude of non-communicable diseases. For example, the elevated levels of circulating cytokines, particularly IL-6, stimulate the liver to produce acute-phase reactants. The most notable of these is C-reactive protein (CRP), a highly sensitive marker of systemic inflammation and a powerful independent predictor of future cardiovascular events [36]. Thus, the inflamed periodontium acts as a persistent source of inflammatory signals that can prime and perpetuate pathological processes in distant organs, such as the vascular endothelium.

However, it is imperative to distinguish between epidemiological associations and definitive causality. While the mechanistic pathways discussed are biologically plausible, much of the current evidence, particularly regarding Alzheimer’s Disease and metabolic syndrome, relies on cross-sectional data or animal models [37,38]. Confounding variables such as socioeconomic status, smoking, and shared dietary habits present significant challenges in isolating the independent contribution of the oral microbiome. Acknowledging these limitations is crucial, as many observed links may be correlational rather than causal, requiring further validation through long-term human longitudinal studies.

### Molecular mimicry and autoimmunity: the role of microbial antigens in breaking self-tolerance

One of the most sophisticated mechanisms linking the oral microbiome to systemic disease involves the triggering of autoimmunity through molecular mimicry and the generation of novel antigens. This pathway is most extensively documented in the pathogenesis of Rheumatoid Arthritis (RA), a systemic autoimmune disease characterised by chronic joint inflammation. The link revolves around a post-translational modification process called citrullination, where the amino acid arginine is converted to citrulline [39].

In genetically susceptible individuals, the immune system can lose tolerance to self-proteins that have been citrullinated, leading to the production of highly specific autoantibodies known as anti-citrullinated protein antibodies (ACPAs), which are the serological hallmark of RA [40]. The oral microbiome plays a unique and central role in this process. The keystone oral pathogen.

*Porphyromonas gingivalis* is exceptional in that it is the only known human pathogen to express its own functional peptidylarginine deiminase (PAD) enzyme, termed PPAD [41]. This bacterial enzyme can directly citrullinate both its own proteins and host proteins within the periodontal tissues, creating a diverse array of neo-antigens that can initiate an ACPA response [42].

Furthermore, other oral pathogens can contribute to this process indirectly. *Aggregatibacter actinomycetemcomitans*, for instance, produces a leukotoxin (LtxA) that lyses neutrophils. This lysis releases the neutrophils' intracellular contents, including the human PAD enzymes (PAD2 and PAD4), leading to a state of ‘hypercitrullination’ in the local environment [43].

This concept has been powerfully substantiated by recent groundbreaking research. A 2023 study demonstrated that RA patients with active periodontitis experience repeated episodes of bacteremia involving a broad range of oral bacteria that have been citrullinated *in situ* within the oral cavity [44]. These circulating citrullinated bacteria are then targeted by highly specific, somatically hypermutated ACPAs found in the patients' blood. This provides a direct, mechanistic link between a breach in the oral mucosal barrier, the systemic dissemination of citrullinated microbial antigens, and the activation and affinity maturation of the B cells responsible for producing RA-specific autoantibodies. This transforms the understanding of RA pathogenesis from a single ‘hit’ event to a chronic disease process that is continually re-stimulated by an ‘antigenic load’ originating from the oral mucosa. This finding suggests that aggressive management of periodontitis could be a crucial disease-modifying strategy in RA, not merely the management of a comorbidity.

### The metabolic connection: microbial metabolites and host metabolic dysregulation

The influence of the oral microbiome is not limited to the translocation of whole bacteria or inflammatory molecules; it also extends to the systemic effects of microbial metabolites. These small molecules, produced during bacterial metabolism, can enter the circulation and interact with host metabolic pathways in distant organs.

A prominent example is trimethylamine *N*-oxide (TMAO). TMAO is a pro-atherogenic metabolite that has been strongly linked to an increased risk of cardiovascular disease. While primarily characterised as a product of gut microbial metabolism, emerging evidence suggests that dysbiotic oral bacteria may also contribute to the systemic TMAO pool, potentially through the swallowing of oral pathobionts which then alter the metabolic output of the gut microbiota [45,46]. This discovery provides another direct biochemical link between oral dysbiosis and cardiovascular risk, independent of direct bacterial invasion or systemic inflammation.

The metabolic link is also evident in Type 2 Diabetes Mellitus (T2DM). Studies using metabolomics have revealed that patients with T2DM have distinct metabolite profiles in their saliva and supragingival plaque compared to healthy individuals. These changes include significantly elevated levels of metabolites such as cadaverine in saliva and *N*-acetyldopamine in plaque [47]. Importantly, the levels of these metabolites correlate with the abundance of specific oral microorganisms, suggesting that the altered metabolic state of T2DM selects for a microbial community with a distinct metabolic output, which may in turn further contribute to systemic metabolic dysregulation.

The interplay between these mechanisms reveals a self-perpetuating pathological network. For example, bacteremia from *P. gingivalis* (Mechanism 4.1) introduces LPS into the bloodstream. This LPS triggers a systemic inflammatory response (Mechanism 4.2), elevating CRP and pro-inflammatory cytokines [48]. This systemic inflammation can then increase insulin resistance (Mechanism 4.4) and promote endothelial dysfunction, which accelerates the development of atherosclerosis [29]. Concurrently, the same pathogen, *P. gingivalis*, can trigger a highly specific autoimmune response via citrullination (Mechanism 4.3) in a susceptible host [49]. This illustrates how a single dysbiotic state in the oral cavity can initiate multiple, parallel pathological cascades that converge to promote and sustain systemic disease. This interconnectedness implies that clinical interventions targeting one pathway, such as reducing the oral inflammatory burden, could have beneficial ripple effects across the entire network (Table 1).

**Table 1.** Key oral pathobionts and their systemic impact.

Pathobiont	Key virulence factors	Primary systemic disease association	Key mechanistic link
*Porphyromonas gingivalis*	Gingipains, Lipopolysaccharide (LPS), Fimbriae, Peptidylarginine Deiminase (PPAD)	Rheumatoid Arthritis (RA), Cardiovascular Disease (CVD), Alzheimer's Disease (AD), Cancer	Protein citrullination, systemic inflammation, endothelial dysfunction, blood-brain barrier disruption, oncogenic signalling
*Fusobacterium nucleatum*	FadA and Fap2 adhesins, LPS	Colorectal Cancer (CRC), Oral Cancer, Inflammatory Bowel Disease (IBD), Adverse Pregnancy Outcomes	Immune evasion (TIGIT binding), oncogenic signalling (Wnt/β-catenin), gut dysbiosis, inflammation
*Aggregatibacter actinomycetemcomitans*	Leukotoxin A (LtxA), Cytolethal Distending Toxin (CDT)	Rheumatoid Arthritis (RA), Cardiovascular Disease (CVD)	Neutrophil-mediated hypercitrullination, induction of systemic inflammation
*Treponema denticola*	Major outer sheath protein (Msp), Dentilisin	Cardiovascular Disease (CVD), Alzheimer's Disease (AD)	Endothelial cell invasion, reduced nitric oxide levels, potential neuroinflammation
*Streptococcus mutans/spp.*	Glucosyltransferases (GTFs), Acidogenicity, Aciduricity	Infective Endocarditis, Cardiovascular Disease (CVD)	Induction of platelet aggregation through surface proteins, biofilm formation on endocardial surfaces, and triggering of pro-inflammatory cytokines through transient bacteremia

### The oral-gut axis: a critical interconnection

The oral cavity and the gastrointestinal tract, while harbouring distinct microbial ecosystems, are anatomically continuous and immunologically linked. The concept of the ‘oral-gut axis’ recognises that microbial and inflammatory events in one location can have profound and often bidirectional consequences for the other [50]. This axis represents a critical pathway through which oral dysbiosis can influence not only gut health but also systemic pathologies that are modulated by the gut microbiome, such as metabolic and neurodegenerative diseases.

#### Microbial translocation from the oral cavity to the intestine

Every day, an individual swallows trillions of oral bacteria along with saliva [50]. Under normal physiological conditions, the harsh acidic environment of the stomach and the antimicrobial properties of bile acids in the duodenum form a formidable barrier that prevents most of these oral microbes from colonising the lower gut [51]. However, this barrier is not impregnable and can be compromised under various conditions.

The long-term use of proton pump inhibitors (PPIs), which suppress gastric acid production, is a well-documented factor that facilitates the survival and intestinal colonisation of oral bacteria [52]. Similarly, conditions associated with achlorhydria (low stomach acid) or impaired bile flow can weaken this barrier. Furthermore, in the context of systemic diseases like inflammatory bowel disease (IBD) or liver cirrhosis, the intestinal environment itself is altered, making it more hospitable to colonisation by ectopic oral microbes [53]. As a result, numerous studies have documented the significant enrichment of typically oral bacterial taxa, such as *Fusobacterium*, *Porphyromonas*, *Veillonella*, and *Klebsiella*, in the gut microbiomes of patients with IBD, colorectal cancer, and chronic liver disease [54].

#### Impact of oral bacteria on gut microbiome composition and intestinal barrier function

The arrival and colonisation of oral pathobionts in the intestine can have destabilising effects on the resident gut microbial community. These oral bacteria can outcompete beneficial commensals, leading to a state of intestinal dysbiosis that mirrors the dysbiosis in the oral cavity [55]. This disruption can alter the metabolic output of the gut microbiome, for example, by reducing the production of beneficial short-chain fatty acids (SCFAs).

Perhaps more critically, certain oral pathogens have been shown to directly compromise the integrity of the intestinal epithelial barrier. For instance, animal studies have demonstrated that oral administration of *P. gingivalis* can increase intestinal permeability, a condition often referred to as ‘leaky gut’ [53]. A compromised intestinal barrier allows for the translocation of microbial products, such as LPS, from the gut lumen into the systemic circulation. This process, known as metabolic endotoxemia, is a powerful driver of the low-grade systemic inflammation associated with metabolic diseases like obesity and T2DM.

This interplay establishes a complex, multi-organ network. For example, the ‘oral-gut-brain axis’ posits that oral dysbiosis can influence neuroinflammatory processes not only through direct hematogenous spread but also indirectly by first altering the gut microbiome and increasing intestinal permeability, which in turn modulates neuro-immune signalling [56]. The evidence for a bidirectional relationship between periodontitis and IBD further supports the concept of a unified mucosal immune system, where an inflammatory response initiated in the mouth can prime immune cells that then contribute to pathology in the gut, and vice versa [57]. This interconnectedness implies that effective treatment for diseases like IBD may require a dual focus on restoring homoeostasis in both the oral and gut ecosystems to break the chronic cycle of inflammation.

## The oral-systemic disease axis: evidence from major pathologies

The mechanistic pathways described above provide the biological framework for understanding how oral dysbiosis translates into systemic disease. This section delves into the specific evidence linking the oral microbiome to a range of major chronic pathologies, highlighting the clinical associations and the underlying molecular interactions that define each disease axis.

### Cardiovascular diseases: the link between periodontitis, atherosclerosis, and hypertension

The association between oral health, particularly periodontitis, and atherosclerotic cardiovascular disease (ASCVD) is one of the most extensively studied and well-supported examples of the oral-systemic link [58]. A substantial body of epidemiological evidence demonstrates that individuals with periodontitis have a significantly increased risk of developing coronary artery disease, suffering a myocardial infarction, and experiencing an ischaemic stroke [59]. This association is not merely a correlation due to shared risk factors like smoking; a 2024 umbrella review that synthesised the findings of 41 separate systematic reviews reaffirmed a significant and independent relationship between periodontal disease and CVD [60].

The mechanisms underpinning this connection are multifaceted, consistent with the pathways outlined previously.

Direct Vascular Invasion and Inflammation: A primary mechanism involves the direct action of oral pathogens on the vascular system. Following bacteremia, bacteria like *P. gingivalis* can directly invade and colonise the endothelial cells of blood vessels, as well as atherosclerotic plaques themselves [61]. The presence of these pathogens within the plaque contributes to local inflammation, promoting the recruitment of immune cells, the proliferation of smooth muscle cells, and the overall progression of the atheroma [61]. Recent primary research continues to solidify this link; a 2023 study identified specific oral bacteria, *Streptococcus anginosus* and *Streptococcus oralis*, in saliva and found their abundance correlated directly with coronary artery calcium scores, a direct measure of atherosclerotic burden [62].

Systemic Inflammatory Burden: Beyond direct invasion, the chronic systemic inflammation originating from the periodontium plays a pivotal role. The continuous influx of pro-inflammatory cytokines (TNF-α,IL-6) and bacterial LPS into the circulation promotes a systemic pro-atherogenic state [63]. This systemic inflammation leads to endothelial dysfunction—a critical early event in atherosclerosis—by reducing the bioavailability of the vasodilator nitric oxide (NO) and increasing the expression of adhesion molecules that facilitate the recruitment of leucocytes into the vessel wall.

Metabolic Dysregulation: The oral microbiome also influences CVD risk through metabolic pathways. Oral dysbiosis has been linked to dyslipidemia, characterised by increased levels of LDL cholesterol and triglycerides [64]. Furthermore, the production of the pro-atherogenic metabolite TMAO by oral bacteria provides another direct biochemical link to cardiovascular risk [65].

Hypertension: The role of the oral microbiome in regulating blood pressure is an emerging area of intense interest. The oral cavity is a primary site for the conversion of dietary nitrate into nitrite by commensal bacteria. This nitrite is then absorbed and can be converted to NO in the circulation, which is a potent vasodilator crucial for maintaining vascular homoeostasis and healthy blood pressure [29]. Oral dysbiosis can lead to a depletion of these beneficial nitrate-reducing bacteria, thereby impairing this pathway, reducing NO bioavailability, and potentially contributing to the development of hypertension [66]. Recent studies have indeed found significant differences in the oral microbial composition of hypertensive individuals compared to normotensive controls, supporting this mechanistic hypothesis [67].

### Metabolic disorders: the bidirectional relationship with type 2 diabetes mellitus (T2DM)

**Figure 2.**
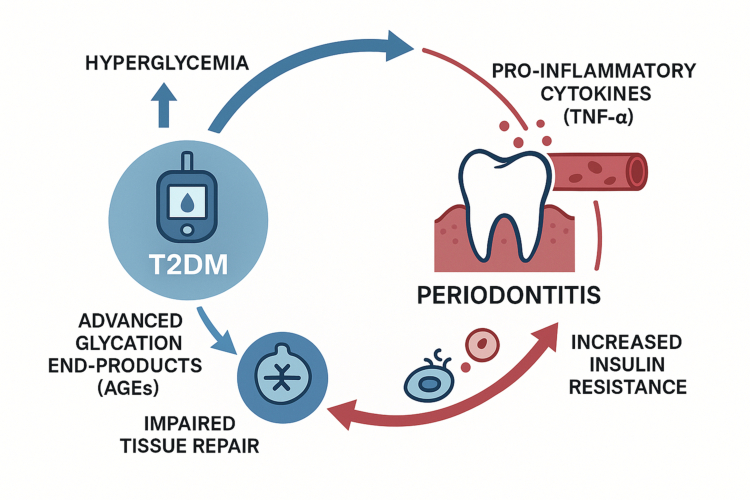
The bidirectional and cyclical relationship between Type 2 Diabetes Mellitus (T2DM) and periodontitis. This schematic illustrates the vicious cycle connecting the two conditions. On one path (blue arrows), T2DM promotes periodontitis; the state of hyperglycaemia leads to the formation of advanced glycation end-products (AGEs), which amplify local inflammation and result in impaired tissue repair. Conversely (red arrows), periodontitis exacerbates T2DM; chronic oral inflammation leads to the systemic release of pro-inflammatory cytokines such as TNF-*α*, which interfere with insulin signalling and cause increased systemic insulin resistance, thereby worsening glycemic control.

The relationship between periodontitis and Type 2 Diabetes Mellitus (T2DM) is a classic example of a bidirectional, or two-way, pathological link [68]. It is well-established that T2DM is a significant risk factor for the development and severity of periodontal disease. The hyperglycemic state in diabetic patients leads to the formation of advanced glycation end products (AGEs), which amplify inflammatory responses and impair tissue repair. Furthermore, elevated glucose levels in the gingival crevicular fluid create a favourable environment for the growth of pathogenic bacteria, promoting a dysbiotic shift [69] (Figure 2).

Conversely, and perhaps more significantly from a systemic perspective, severe periodontitis can adversely affect glycemic control and increase the risk of developing diabetic complications [70]. The primary mechanism is the chronic systemic inflammation emanating from the diseased periodontium. The elevated levels of circulating pro-inflammatory cytokines, particularly TNF-α, interfere with insulin signalling pathways, thereby exacerbating systemic insulin resistance—the core pathophysiological defect in T2DM [71]. This creates a vicious cycle where diabetes promotes periodontitis, and periodontitis, in turn, worsens diabetes.

A critical finding that underscores the depth of this connection is that the oral microbiome is altered in T2DM patients even in the absence of clinically diagnosed oral disease. This suggests that the oral microbiome may serve as an early and sensitive indicator of underlying systemic metabolic dysregulation. Metagenomic sequencing studies of orally healthy T2DM patients have revealed a distinct microbial signature characterised by a significant enrichment of periodontal pathobionts, such as *Porphyromonas gingivalis* and *Prevotella melaninogenica*, compared to healthy controls [72]. This microbial shift is accompanied by a corresponding alteration in the oral metabolome, with increased levels of metabolites like cadaverine and *N*-acetyldopamine [72]. This temporal insight—that microbial and metabolic changes in the mouth precede overt clinical pathology—is profound. It reframes the oral microbiome not just as a consequence of established disease, but as a real-time barometer of systemic physiological stress. This opens up exciting possibilities for preventative medicine, where routine analysis of saliva could potentially identify individuals at high risk for T2DM long before conventional markers like HbA1c become abnormal. However, it is worth noting that the evidence is not entirely uniform; some studies have found no significant differences in overall alpha-diversity between T2DM and control groups, suggesting that specific compositional or functional shifts, potentially influenced by shared factors like diet and lifestyle, are more critical than simple diversity metrics [73] (Figure 2).

### Neurodegenerative conditions: the oral-brain axis in Alzheimer's disease (AD)

**Figure 3.**
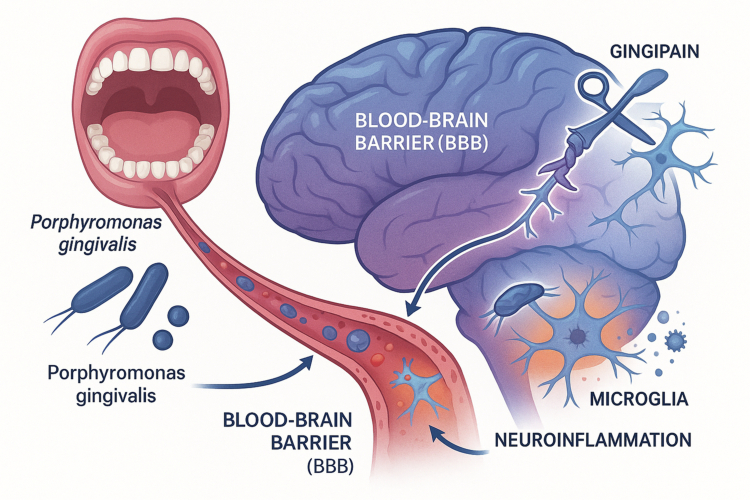
The ‘oral-brain axis’ and the proposed role of Porphyromonas gingivalis in Alzheimer's disease (AD) pathology. The diagram illustrates the translocation of the oral pathogen *P*. gingivalis from the oral cavity to the brain via hematogenous dissemination. The pathogen and its virulence factors can compromise and cross the blood-brain barrier (BBB). Once inside the central nervous system, *P*. gingivalis can trigger pathological events central to AD. This includes the activation of resident immune cells (microglia), leading to a state of chronic neuroinflammation, and the release of neurotoxic gingipain proteases, which are implicated in neuronal damage.

The ‘infectious hypothesis’ of Alzheimer's Disease (AD), which posits that microbial agents may play a role in initiating or propagating the neurodegenerative process, has gained considerable traction in recent years. Within this framework, a compelling body of evidence has emerged implicating oral pathogens, with *Porphyromonas gingivalis* being the most studied culprit [74]. The concept of an ‘oral-brain axis’ is now supported by multiple lines of mechanistic evidence (Figure 3).

Direct Brain Invasion and Neurotoxicity: The most direct mechanism involves the translocation of oral pathogens or their virulence factors into the central nervous system. Post-mortem studies have identified *P. gingivalis* DNA and its key virulence factors, the gingipain proteases, within the brain tissue of AD patients [75]. These pathogens can cross a compromised blood-brain barrier (BBB), which can itself be made more permeable by the systemic inflammation originating from periodontitis [76]. Once in the brain, gingipains have been shown to be neurotoxic and can cleave the tau protein, a process associated with the formation of neurofibrillary tangles, one of the pathological hallmarks of AD [76].

Neuroinflammation: Systemic inflammation driven by oral dysbiosis is another critical pathway. Circulating pro-inflammatory cytokines and bacterial LPS can cross the BBB and activate the brain's resident immune cells, the microglia and astrocytes [77]. In AD, these cells enter a state of chronic activation, releasing neurotoxic substances and driving a self-perpetuating cycle of neuroinflammation that contributes to neuronal damage and cognitive decline [78].

Modulation of AD Pathology: Oral pathogens appear to directly interact with the core pathological proteins of AD. The presence of *P. gingivalis* in animal models is associated with an increased load of amyloid-beta (Aβ) plaques [75]. An intriguing related hypothesis suggests that Aβ itself may function as an antimicrobial peptide (AMP), and its accumulation could represent a protective, albeit ultimately pathological, response to chronic microbial invasion of the brain [49]. Other oral bacteria, such as spirochaetes from the genus *Treponema*, have also been detected in AD brains and may contribute to pathology, potentially using cranial nerves like the trigeminal nerve as a direct route of entry [74].

### Autoimmune disorders: oral pathogens and the etiopathogenesis of rheumatoid arthritis (RA)

As detailed in Section 4.3, the mechanistic link between oral dysbiosis and Rheumatoid Arthritis (RA) is one of the most compelling and well-elucidated examples of the oral-systemic connection [49]. The central pillar of this association is the process of protein citrullination and the subsequent generation of ACPAs, the highly specific autoantibodies that drive RA pathogenesis [79].

The oral cavity is now considered a primary site for the initial break in immune tolerance that leads to RA. As elaborated in Section 4.3, the citrullination of host and bacterial proteins is a pivotal event. *P*. gingivalis contributes directly via its PPAD enzyme, while the inflammatory milieu, exacerbated by pathogens such as A. actinomycetemcomitans, promotes neutrophil-mediated hypercitrullination [80]. This creates a rich source of potential autoantigens that, in a genetically predisposed individual, can trigger the production of ACPAs years before the clinical onset of arthritis [81].

The oral microbiome of RA patients is distinctly different from that of healthy individuals, showing an enrichment of taxa such as *Prevotella* and *Leptotrichia* and a depletion of *Haemophilus* [79]. Furthermore, the oral-gut axis (discussed in Section 4.5) plays a critical role. Oral pathogens swallowed from the dysbiotic oral cavity can translocate to the gut, where they can alter the intestinal microbiome and promote the differentiation of pro-inflammatory T helper 17 (Th17) cells. These pathogenic Th17 cells can then migrate from the gut to the joints, where they contribute directly to synovial inflammation and bone destruction [82].

### Oncogenesis: the role of oral bacteria in oral and gastrointestinal cancers

**Figure 4.**
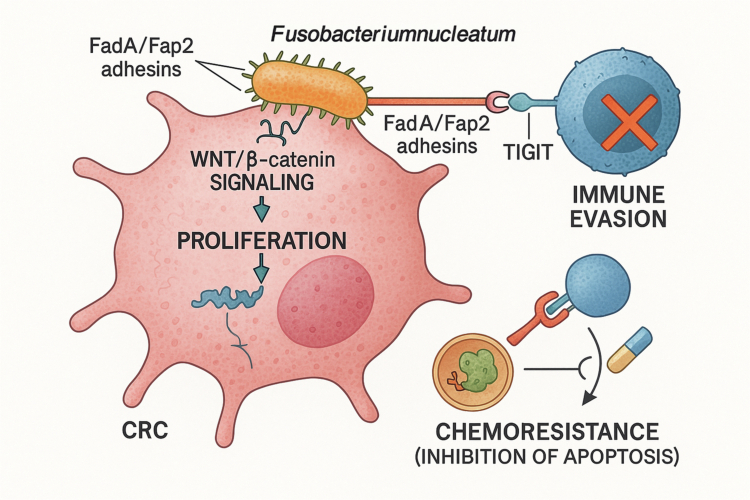
Mechanisms of Fusobacterium nucleatum in promoting colorectal cancer (CRC) progression. The oral bacterium F. nucleatum utilises its FadA/Fap2 adhesins to specifically colonise CRC cells. Once established, it employs several mechanisms to drive oncogenesis. (1) Proliferation: It directly activates the intracellular Wnt/β-catenin signalling pathway, a key driver of tumour cell proliferation in CRC. (2) Immune evasion: Its Fap2 protein binds to the inhibitory TIGIT receptor on immune cells (e.g. T cells and NK cells), suppressing their anti-tumour activity. (3) Chemoresistance: It modulates host cellular processes, such as autophagy, to protect cancer cells from chemotherapy-induced apoptosis.

The long-established link between chronic inflammation and cancer has led researchers to investigate the role of the oral microbiome in carcinogenesis. It is now clear that specific oral bacteria, particularly *Fusobacterium nucleatum* and *Porphyromonas gingivalis*, can act as ‘oncomicrobes,’ actively contributing to the development and progression of certain malignancies [83] (Figure 4).

Oral Squamous Cell Carcinoma (OSCC): As the local microbiome, it is not surprising that oral bacteria are implicated in OSCC. Both *P. gingivalis* and *F. nucleatum* are found to be enriched in OSCC tissues. They are believed to promote tumorigenesis by creating a chronic inflammatory microenvironment, stimulating cell proliferation through pathways like IL-6-STAT3, and inhibiting apoptosis, thereby allowing malignant cells to survive and grow [84].

Colorectal Cancer (CRC): The role of *F. nucleatum* in CRC is a paradigm-shifting discovery. This bacterium, typically a resident of the oral cavity, can translocate to the lower gastrointestinal tract, where it preferentially colonises colorectal tumours [53]. Its FadA and Fap2 adhesins allow it to bind specifically to CRC cells [85]. Once established, it is not a passive bystander but an active participant in cancer progression. This elevates the bacterium from a simple inflammatory trigger to a sophisticated co-conspirator in carcinogenesis. This understanding has direct therapeutic implications, as it suggests that CRC patients with high *F. nucleatum* loads may benefit from adjuvant therapies that target the bacterium itself, potentially enhancing the efficacy of conventional treatments like immunotherapy.

The mechanisms employed by *F. nucleatum* are remarkably sophisticated:

Promotion of Oncogenic Signalling: It directly activates the Wnt/β-catenin signalling pathway, a core pathway in CRC, to drive tumour cell proliferation [86].

Immune Evasion: *F. nucleatum* creates an immunosuppressive tumour microenvironment to protect the tumour from the host immune system. Its Fap2 protein binds to the inhibitory TIGIT receptor on T cells and Natural Killer (NK) cells, effectively paralyzing their anti-tumour activity [87]. In a further layer of immune sabotage, recent research has shown that *F. nucleatum* releases a metabolite, ADP-heptose, which activates an intracellular signalling pathway (via the ALPK1 receptor) in cancer cells. This activation upregulates PD-L1 on tumour surfaces. Consequently, PD-L1 binds to PD-1 receptors on T cells, directly inducing their exhaustion and aiding tumour survival [88].

Chemoresistance: It has been shown to confer resistance to chemotherapy by modulating cellular processes like autophagy and inhibiting apoptosis in cancer cells [89].

## Clinical perspectives and future horizons

The growing body of evidence firmly establishing the oral microbiome as a modulator of systemic health has profound clinical implications. This knowledge is paving the way for novel diagnostic approaches, innovative therapeutic strategies, and a necessary paradigm shift towards a more integrated model of healthcare. However, the translation of these discoveries from the laboratory to the clinic is contingent upon addressing significant methodological challenges within the field.

### The oral microbiome as a diagnostic and prognostic biomarker

The oral cavity offers a distinct advantage for biomarker discovery: it is easily accessible, and sample collection, primarily of saliva, is non-invasive, simple, and can be performed repeatedly [3]. This makes the oral microbiome an exceptionally attractive target for the development of diagnostic, prognostic, and monitoring tools for a wide range of systemic diseases [90].

Research is rapidly moving in this direction. Studies conducted in large, healthy populations have already demonstrated that the composition of the oral microbiome correlates with key systemic clinical biomarkers, such as fasting glucose, insulin levels, and white blood cell counts [90]. This suggests that even in a state of apparent health, the oral microbiome can reflect an individual's underlying metabolic and inflammatory status, potentially serving as an early warning system for disease risk.

More specifically, distinct microbial and metabolomic signatures are being identified for various pathologies. For example, recent integrated analyses have shown that patients with acute myocardial infarction (AMI) have a characteristic oral microbiome enriched in genera like *Streptococcus* and *Rothia*, alongside a unique salivary metabolomic profile [91]. Similarly, specific microbial signatures have been proposed for oral squamous cell carcinoma [92] and T2DM [72]. The ultimate goal is to leverage these signatures to develop robust, validated clinical tests that could be used for population screening, early diagnosis, or predicting disease progression and treatment response.

However, a significant hurdle remains. Methodological rigour is currently the primary bottleneck preventing the widespread clinical translation of these findings. Many of the associations reported to date are derived from small, cross-sectional studies with inconsistent sampling protocols and insufficient control for confounding variables [93]. While these studies are crucial for hypothesis generation, the biomarkers they identify must be rigorously validated in large-scale, multi-centre, longitudinal cohort studies. Without such validation, the promise of oral microbiome-based diagnostics will remain unrealised. This represents a critical call to action for the research community to prioritise the establishment of standardised methodologies and to invest in the large, well-designed studies needed to bring these promising tools to clinical practice.

### Therapeutic strategies: modulating the oral microbiome for systemic health

The understanding that oral dysbiosis contributes to systemic disease opens up a new therapeutic frontier: targeting the oral microbiome to improve overall health. These strategies range from reinforcing conventional approaches to developing highly specific, next-generation interventions.

#### Conventional approaches: hygiene, diet, and probiotics

The foundation for maintaining oral eubiosis remains the consistent practice of good oral hygiene, including regular brushing and interdental cleaning, to control the accumulation of plaque biofilm [94]. This is complemented by lifestyle and dietary modifications, particularly the reduction of dietary sugars that fuel cariogenic dysbiosis [21].

Beyond these basics, several adjunctive therapies are available. The use of sugar alcohols like xylitol has been shown to reduce the levels of *S. mutans* by interfering with its metabolism [95]. Various antimicrobial oral rinses containing agents such as cetylpyridinium chloride (CPC), essential oils, or stabilised chlorine dioxide can help to reduce the overall bacterial load and control gingivitis [96].

A more ecologically-minded approach involves the use of probiotics—live beneficial bacteria that can help to restore a healthy microbial balance. Specific strains, such as *Lactobacillus reuteri* and *Streptococcus salivarius* K12 and M18, have been studied for their ability to competitively exclude pathogens, modulate local inflammation, and promote oral health [97]. These interventions represent the first steps towards a therapeutic philosophy focused on ‘ecological engineering’ rather than simple bacterial eradication.

#### Emerging therapies: phage therapy and precision interventions

The future of oral microbiome therapeutics lies in precision medicine, with strategies designed to selectively target pathogenic species while preserving the beneficial commensal community. Bacteriophage (phage) therapy is at the forefront of this new paradigm [98]. Phages are viruses that infect and kill bacteria with extremely high specificity. This allows for the targeted elimination of a single pathogenic species, such as *P. gingivalis*, without causing collateral damage to the surrounding ecosystem [99]. In the context of periodontitis, the goal of phage therapy would be to induce the collapse of the dysbiotic biofilm community, thereby resolving inflammation and allowing a healthy, eubiotic community to re-establish itself [99]. Genetically engineered phages are also being developed to enhance their therapeutic potential, for example, by equipping them with enzymes that can degrade the protective matrix of biofilms or by using them to deliver gene-editing tools like CRISPR-Cas to disable antibiotic resistance genes in target bacteria [100]. Phage therapy also holds promise as an adjuvant in cancer treatment, where it could be used to eliminate oncomicrobes like *F. nucleatum* from the tumour microenvironment [101].

Another precision approach involves targeting specific virulence factors. For example, small-molecule inhibitors of the gingipain proteases of *P. gingivalis* have been developed. By neutralising this key virulence factor, these inhibitors can disarm the pathogen, reducing its ability to cause tissue destruction and manipulate the host immune response. These compounds are currently being investigated in clinical trials for their potential to treat not only periodontitis but also AD, given the role of gingipains in neuroinflammation [102]. These highly targeted strategies embody a shift towards restoring ecological balance rather than indiscriminate microbial killing, offering a more sustainable and potentially more effective approach to managing dysbiosis-related diseases (Table 2).

**Table 2.** Summary of therapeutic interventions to modulate the oral microbiome.

Intervention	Mechanism of action	Primary target/application	Level of evidence/status
Probiotics (*L. reuteri*, *S. salivarius*)	Competitive exclusion, pH modulation, production of bacteriocins, immune modulation	Caries/periodontitis prevention, halitosis, restoration of eubiosis	Clinical studies show benefit for specific endpoints
Prebiotics/Dietary (Xylitol, Fibre)	Inhibition of pathogenic metabolism (*S. mutans*), promotion of beneficial species	Caries prevention (Xylitol)	Well-established in clinical studies and practice
Oral Rinses (CPC, Essential Oils)	Broad-spectrum antimicrobial activity, disruption of bacterial cell membranes	General plaque control, gingivitis management	Widely used, strong clinical evidence for efficacy
Phage Therapy	Highly specific, lytic infection and killing of target bacterial species	Precision treatment of periodontitis, anti-cancer adjuvant (e.g. targeting *F. nucleatum*)	Pre-clinical and early clinical trial stage; highly promising
Gingipain Inhibitors	Specific inhibition and neutralisation of a key virulence factor of *P. gingivalis*	Periodontitis, Alzheimer's Disease (AD)	Pre-clinical and clinical trial stage for both oral and systemic indications

### Methodological challenges and the path forward in oral microbiome research

Despite the rapid progress and immense potential of the field, several key methodological challenges must be addressed to ensure the reliability, reproducibility, and ultimate clinical translation of oral microbiome research.

Standardisation of Sampling: The oral cavity is a mosaic of different microbial habitats. The microbial profile obtained from a saliva sample can differ significantly from that of a subgingival plaque sample or a mucosal swab from the same individual. Currently, there is a lack of standardised protocols for sample collection, processing, and storage, which introduces significant heterogeneity and makes it difficult to compare results across studies [10].

Study Design and Confounding Factors: The majority of existing studies are cross-sectional, which can identify associations but cannot establish causality. There is a critical need for more large-scale, prospective, longitudinal cohort studies that can track changes in the oral microbiome over time and correlate them with disease onset and progression [93]. Furthermore, the oral microbiome is influenced by a vast number of confounding variables, including diet, hygiene practices, socioeconomic status, medication use, and host genetics. Adequately controlling for these factors is a major challenge that requires meticulous study design and sophisticated statistical analysis [103].

From Composition to Function: Most research to date has focused on characterising the taxonomic composition of the microbiome (i.e. ‘who is there?’). However, to truly understand its role in health and disease, the field must move towards functional analysis (i.e. ‘what are they doing?’). This requires the integration of multi-omics approaches, including metagenomics (to assess genetic potential), metatranscriptomics (to measure gene expression), proteomics (to identify proteins), and metabolomics (to profile metabolic byproducts). These integrated analyses will provide a much more dynamic and mechanistic picture of host-microbiome interactions [104].

The path forward requires a concerted effort from the research community to establish standardised protocols, build large and diverse patient cohorts for longitudinal study, and embrace integrated multi-omics approaches. Overcoming these challenges will be essential to unlock the full potential of the oral microbiome as a tool for improving human health.

## Conclusion

The evidence synthesised in this review compellingly demonstrates that the oral microbiome functions as a fundamental regulatory hub with far-reaching implications for systemic health. The traditional view of the oral cavity as an isolated domain, where microbial activities are relevant only to local diseases, is now obsolete. Instead, the oral microbiome must be recognised as an integral component of the human superorganism, a dynamic ecosystem whose balance or imbalance can profoundly influence the trajectory of health and disease throughout the body.

We have delineated the intricate and interconnected mechanistic pathways—hematogenous dissemination, systemic inflammation, molecular mimicry, and metabolic disruption—that link oral dysbiosis to a wide spectrum of chronic, non-communicable diseases. While much evidence remains associative, emerging mechanistic data strongly supports causal pathways in conditions such as cardiovascular disease, type 2 diabetes, Alzheimer's disease, rheumatoid arthritis, and colorectal cancer, specific oral pathobionts and their virulence factors have been mechanistically implicated in pathogenesis. The oral cavity, particularly in the context of periodontitis, serves as a persistent reservoir of pathogenic microbes and inflammatory stimuli that can initiate, perpetuate, and exacerbate disease processes in distant organ systems.

This paradigm shift carries with it a clear clinical imperative: the artificial wall separating dentistry from medicine must be dismantled. The integration of oral health surveillance into routine systemic disease prevention and management is no longer an option but a necessity. The oral microbiome offers a readily accessible window into an individual's inflammatory and metabolic state, holding immense potential for the development of non-invasive biomarkers for early risk assessment and diagnosis. Concurrently, the therapeutic horizon is expanding beyond conventional mechanical and antimicrobial approaches towards sophisticated strategies of ‘ecological engineering,’ such as probiotics and phage therapy, which aim to restore oral eubiosis for systemic benefit.

The path forward, however, requires a commitment to rigorous and standardised scientific methodology. The promise of microbiome-based diagnostics and therapeutics can only be realised through large-scale, longitudinal studies that can definitively establish causality and validate biomarkers in diverse populations. By addressing these challenges, the scientific and clinical communities can harness the power of the oral microbiome, transforming our ability to predict, prevent, and treat the most pressing chronic diseases of our time and ushering in a new era of truly holistic and personalised healthcare.

## Supplementary Material

Supplementary Figure 1.png Supplementary Figure 1.png

## Data Availability

Data sharing is not applicable to this article as no datasets were generated or analysed during the current study.
