# Supplementary figures and images for: The oral microbiome as a regulatory hub for systemic health: a systematic review of mechanistic links and clinical implications
Source: J Oral Microbiol. 2026 Mar 3;18(1):2635233. doi: 10.1080/20002297.2026.2635233 (PMC12958392; doi:10.1080/20002297.2026.2635233)

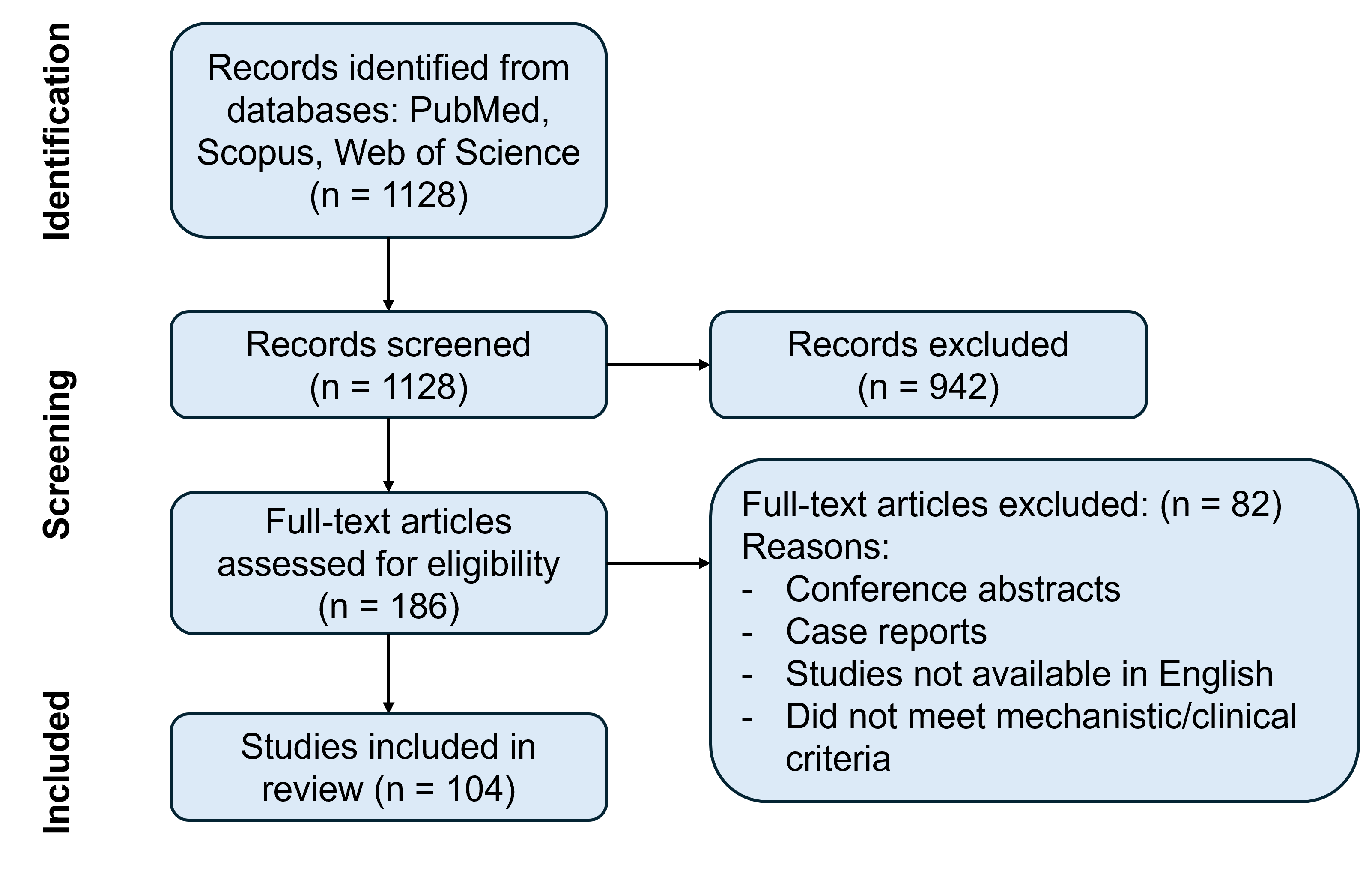

Supplement: Supplementary Figure 1.png [file ZJOM_A_2635233_SM7386.png]
